# Pleomorphic adenocarcinoma of the breast: a case report

**DOI:** 10.3389/fonc.2025.1644881

**Published:** 2025-09-17

**Authors:** Yunkun Zhang, Pengchao Fan, Min Gao, Rui Cong, Qimin Wang, Dan Zhao, Jian Zhang, Yini Zhang, Lifen Wang

**Affiliations:** ^1^ Department of Pathology, The Second Hospital of Dalian Medical University, Dalian, China; ^2^ Hospital Infection Management Department, The Second Hospital of Dalian Medical University, Dalian, China; ^3^ Department of Ultrasonography, The Second Hospital of Dalian Medical University, Dalian, China

**Keywords:** breast, pleomorphic adenocarcinoma, PIK3CA mutation, ultrasound, NGS - next-generation sequencing

## Abstract

This article comprehensively reports a case of pleomorphic adenocarcinoma of the breast, a rare breast salivary gland-type tumor. A mass with a maximum diameter of 3.2 cm was detected through ultrasound within the upper outer quadrant in a 74-year-old woman undergoing left breast total mastectomy and sentinel lymph node exploration. Microscopically, the central part of the tumor was mostly arranged in the solid structures, some of which were cribriform and contained blue-stained secretions, while the periphery of the tumor had striated and glandular duct-like arranged structures. Some tumor cells had visible nucleoli and nuclear fission, with sparse cytoplasm. Immunohistochemistry revealed ER (-), PR (-), HER-2 (0), Bcl-2 (+), P63 (-), Calponin (-), S100 (+), E-cadherin (+), and Ki-67 (20%+). The next-generation sequencing (NGS) assay revealed a missense mutation within exon 7 in PIK3CA, which is related to the treatment of breast cancer. The patient received 1 course of oral Capecitabine treatment after surgery and was followed up for 2 years with no recurrence.

## Introduction

Breast pleomorphic adenocarcinoma represents the uncommon salivary gland-type tumor originating from the breast. To date, only four cases have been reported worldwide. The pathological patterns and immunohistochemical findings vary slightly among cases, but the prognosis differs significantly. Pleomorphic adenocarcinoma of the salivary gland is a low-grade tumor (1), but this conclusion may not hold true in the breast.

This paper is the first to present the large-scale gene sequencing data and identify one missense synonymous mutation within exon 7 of the phosphatidylinositol-3-kinase, catalytic subunit alpha (PIK3CA) gene in this rare disease, which is closely related to breast cancer and its dismal prognosis and treatment (2). In this paper, a comprehensive morphological, immunohistochemical and genetic analysis of this case is presented to further understand the uncommon condition.

## Case report

**Figure 1:**
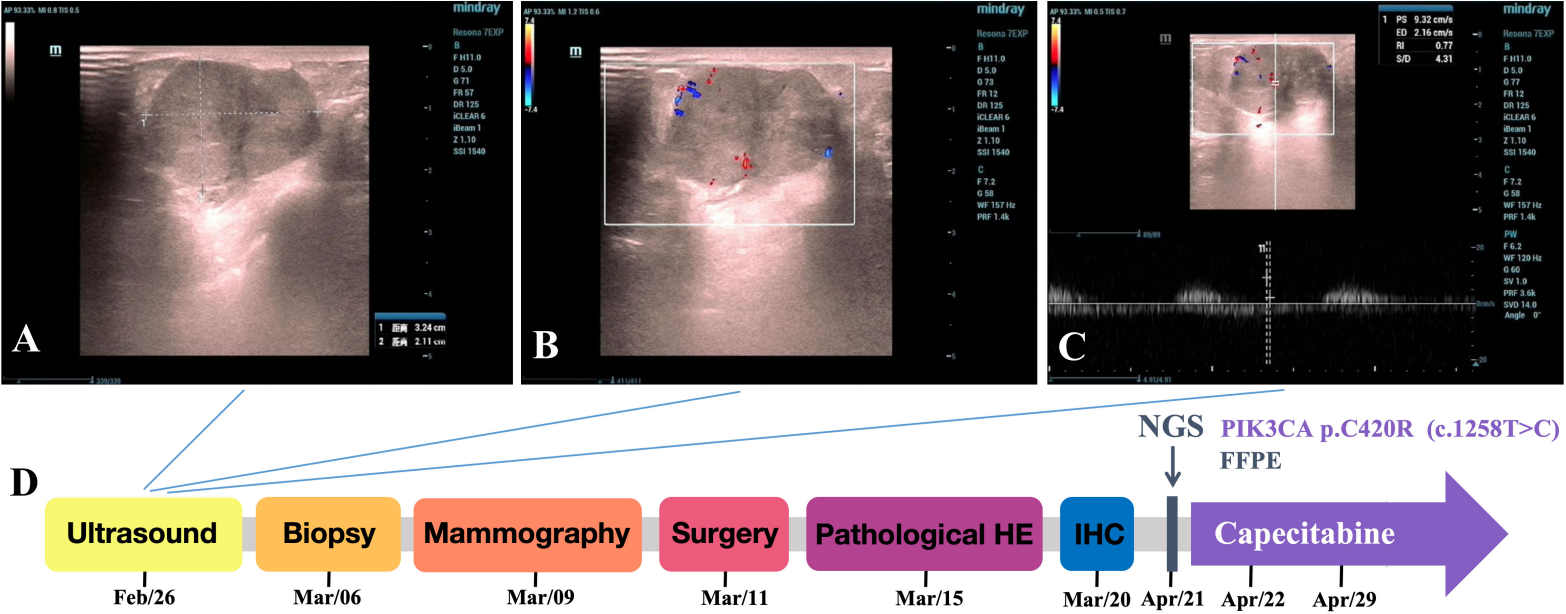
The two-dimensional ultrasound image of the 3.2×2.1 cm hypoechoic mass has relatively clear boundaries, regular morphology, slightly irregular margins, and heterogeneous internal echogenicity. **(B)** The color Doppler ultrasound image showing detectable blood flow signals within the mass. **(C)** The spectral Doppler image demonstrating a resistance index (RI) of 0.77. **(D)** Disease time line showed the various treatment received by the patient.

A 74-year-old woman found a left breast mass and ultrasound examination suggested that a hypoechoic mass measuring 3.2×2.1 cm is observed within the glandular layer. The lesion demonstrates relatively clear boundaries, regular morphology, slightly irregular margins, and heterogeneous internal echogenicity. Color Doppler shows detectable blood flow signals with a Resistive index (RI) of 0.77 (**Figures 1A–C**). Diagnostic conclusion: Solid nodule in left breast (BI-RADS 4A-4B). This patient was sent to our center to receive further treatment. The patient was then subjected to biopsy under local anesthesia, and the pathological finding revealed the presence of carcinoma, which was difficult to classify and was considered a low-grade malignant tumor. Molybdenum digital X-ray radiography examination suggested a mass, and BI-RADS class was 6 (**Figure 1D**). The patient received total mastectomy of the left breast and sentinel lymph node exploration later (**Figure 1D**). The pathological diagnosis was breast pleomorphic adenocarcinoma with no cancer metastasis in the sentinel lymph nodes.

**Figure 2:**
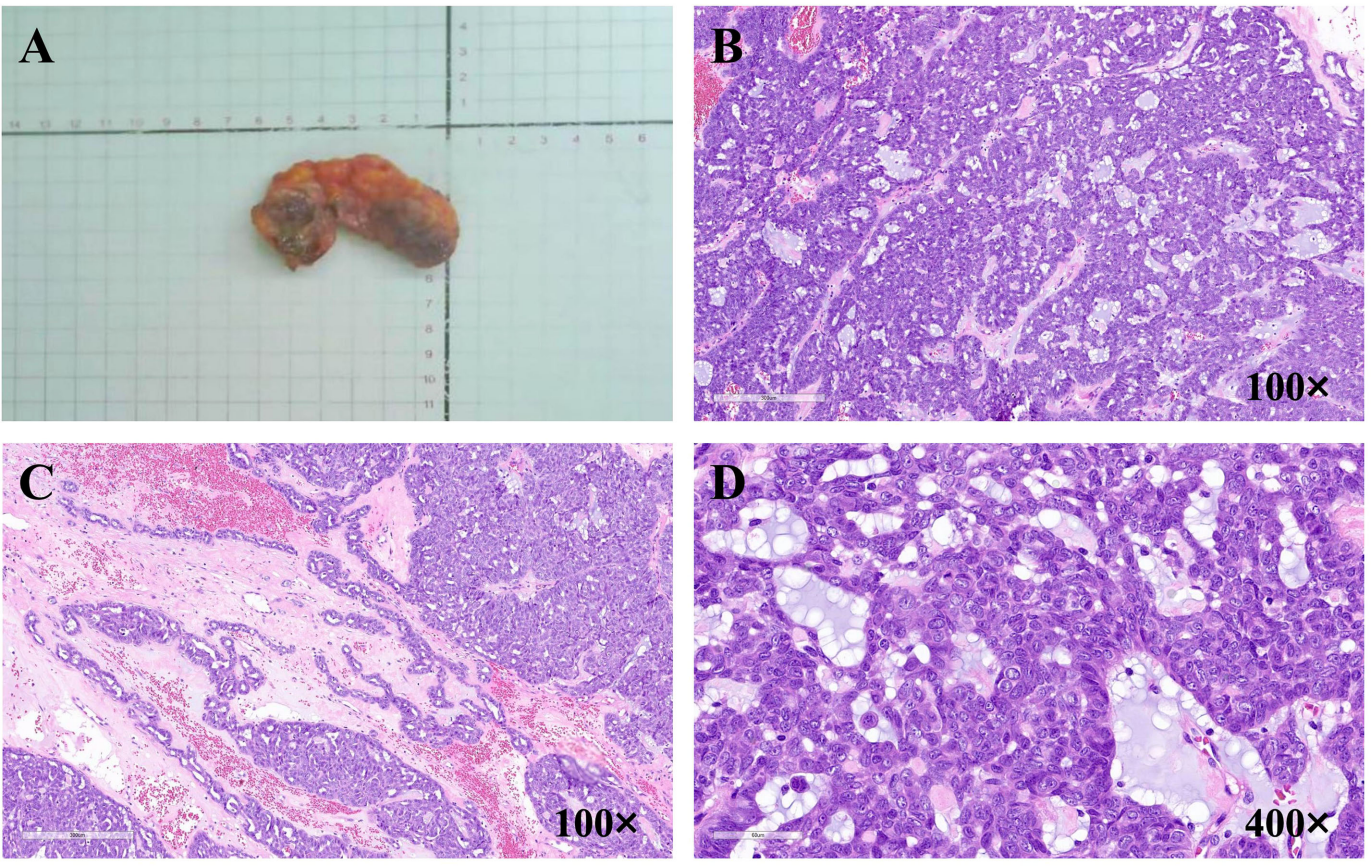
**(A)** The image of tumor gross morphology. **(B)** The image of central part of the tumor microscopically, 100×. **(C)** The image of periphery part of the tumor microscopically, 100×. **(D)** The image of the tumor cells and the mitotic figures, 400×.

The tumor was grayish-white, solid and tough, with poorly defined borders (**Figure 2A**). Microscopically, the central part of the tumor was mostly arranged in solid structures, some of which were cribriform and contained blue-stained secretions (**Figure 2B**), while the periphery of the tumor showed the striated and glandular duct-like arranged structures (**Figure 2C**). The morphology of the tumor cells was consistent with ovoid or fat spindle-shaped nuclei. Some tumor cells had visible nucleoli, with the nuclear fission of about 6–10 highest possible frequency, and the cytoplasm was sparse (**Figure 2D**).

**Figure 3:**
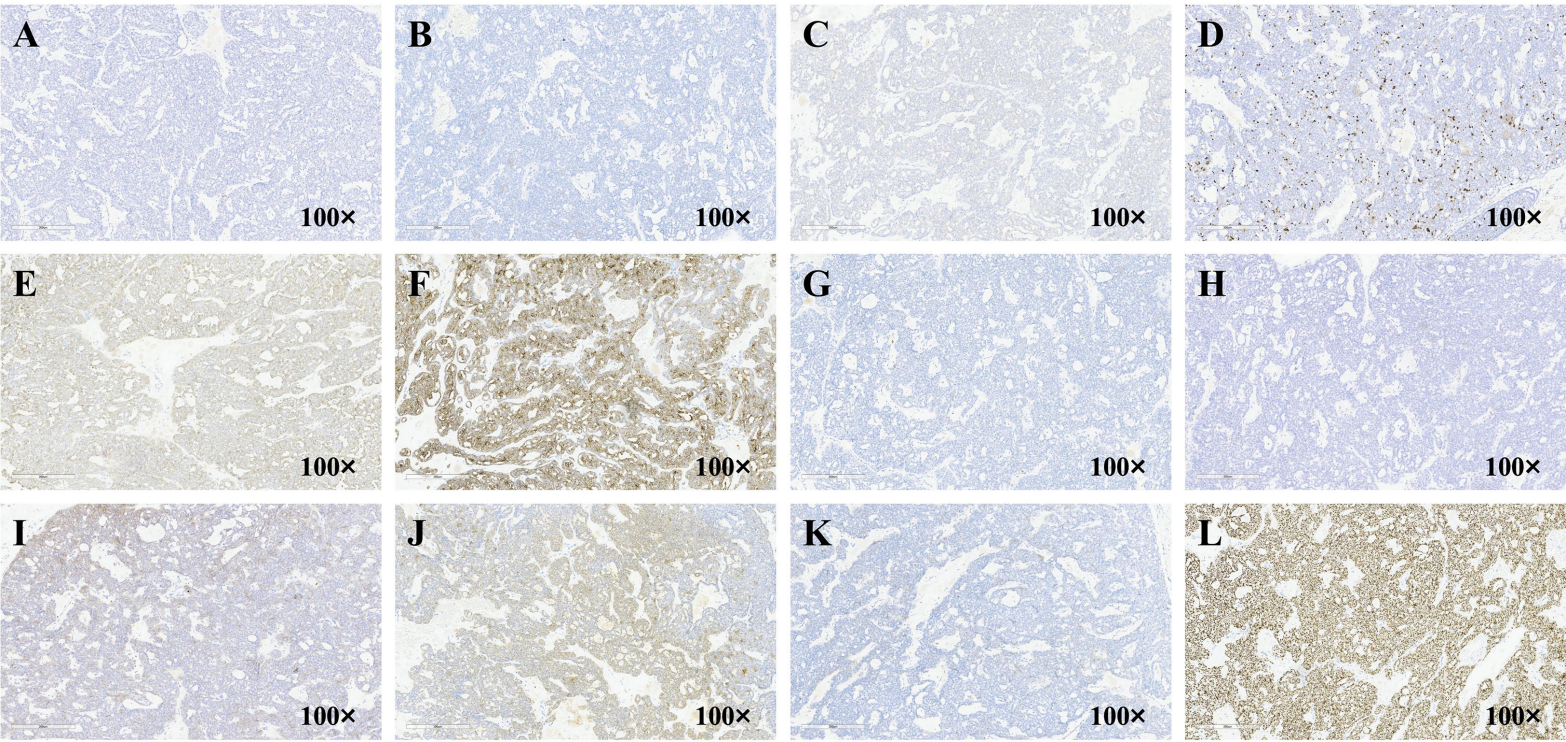
The images of patients’ Immunohistochemical of **(A)** ER, **(B)** PR, **(C)** Her-2, **(D)** ki-67, **(E)** Bcl-2, **(F)** CK7, **(G)** P63, **(H)** Calponin, **(I)** E-cadherin, **(J)** S100, **(K)** CD117, **(L)** SOX-10.

Immunohistochemical results showed estrogen receptor (ER) (-), progesterone receptor (PR) (-), human epidermal growth factor receptor-2 (Her-2) (0), antigen KI-67 (Ki67) (20%+), B-cell lymphoma 2 (Bcl-2) (+), cell keratin 7 (CK7) (focal +), tumor protein 63 (P63) (-), Calponin (-), E-cadherin (+), S100 protein (S100) (+), mast/stem cell growth factor receptor (CD117) (partially +), and SRY-related HMG-box 10 protein (SOX-10) (+) (**Figures 3A–L**).

NGS was conducted using double stranded nucleic acids extracted in the formalin-fixed paraffin-embedded breast tissues. Moreover, targeted NGS was conducted in 425 cancer-associated genes (**Supplementary Table S1**), which gained approval from the College of American Pathologists (CAP) and the Clinical Laboratory Associates (CLA), in Nanjing Geneseeq Technology Co., Ltd. We detected a PIK3CA C420R (c.1258T>C) mutation at the 41.24% mutant allele frequency within the breast lesion, and missense mutations leading to amino acid residue substitutions of the PIK3CA proteins were also detected. More details about genetic alterations can be observed from **Table 1**.

**Table 1:** Analysis of NGS data revealed the following mutations within CDS of the below-mentioned genes.

Gene	CDS mutation	AA mutation	Sequencing depth	Abundance(%)	COSMIC ID	Functional context for mutations
PIK3CA	c.1258T>C	p.C420R	1319	41.24	COSM757	sensitivity of PI3K inhibitors
DPYD	c.85C>T	p.R29C	1066	100	COSM3735988	chemotherapy toxicity
EPCAM	c.344T>C	p.M115T	1336	100	COSM3927762	poor prognosis
XRCC1	c.1196A>G	p.Q399R	516	99.81	COSM3756906	efficacy of chemotherapy
MTHFR	c.665C>T	p.A222V	1157	50.13	COSM146404	chemotherapy toxicity
GSTP1	c.313A>G	p.I105V	793	47.79	COSM3752683	efficacy of chemotherapy
NQO1	c.559C>T	p.P187S	1074	100	COSM148097	poor prognosis of chemotherapy

The woman in our case refused further treatment, so only 1 course of oral Capecitabine was administered after surgery, and the patient was followed up for 2 years with disease-free survival.

## Discussion

Pleomorphic adenocarcinoma of the breast was first described by Asio li et al. in 2006. It is a very rare salivary gland-type tumor originating from the breast, with only four cases being reported so far. In addition to the breast, it has also been occasionally reported within the lung or other tissues (3).

Breast pleomorphic adenocarcinoma occurs mostly in women aged 37–74 years, and tumor sizes may be 1.5–10 cm. From the microscopic perspective, tumor cells are mostly solidly arranged at the center, surrounded by some line-like and tubule-like structures. The tumor cells are round and small, accompanied by centered nuclei, inconspicuous nucleoli, and scanty cytoplasm. Immunohistochemistry suggests that this tumor is a triple-negative breast cancer positive for E-cadherin membrane and Bcl-2, which are all consistent with the results of this paper. It needs to be differentiated from the breast invasive lobular carcinoma, because tumor cells are more uniform in morphology and some of them are arranged in a striated pattern. The present case developed triple-negative breast cancer with E-cadherin positiveness (+), and invasive lobular carcinoma could be excluded. The central morphology of the tumor resembled the adenoid cystic carcinoma, but with no coexistence of myoepithelial and adenoepithelial patterns. In addition, P63 (-) and calponin (-) also indicated the lack of myoepithelium, which did not support the diagnosis of adenoid cystic carcinoma. This patient with triple negative breast cancer showed SOX-10 (+), S-100 (+), and CD117 (partially +). Such expression pattern is similar to that of the secretory carcinoma of the breast, and it has been suggested that CD117 can be positive in pleomorphic adenocarcinoma of the breast (4). However, the morphology of this case was not consistent with secretory carcinoma because of the absence of complex arrangement, cytoplasmic and extracellular eosinophilic secretions. According to literature report, Mamglb, GATA binding protein 3 (GATA-3) and gross cystic disease fluid protein 15 (GCDFP-15) are less effective on proving the origin of salivary gland-type breast tumor, while SOX-10 may be more effective (5). In this case, Mamglb, GCDFP-15 and GATA-3 were negatively expressed and SOX-10 was positively expressed. There was no primary tumor in salivary gland or other locations, so the diagnosis of a primary pleomorphic adenocarcinoma of the breast was confirmed in this case. Because of the extremely few cases, there is still a lot of confusions in the analysis of the immunohistochemical results, and more information is needed to make a correct diagnosis in combination with other clinical information.

PIK3CA gene is a proto-oncogene, which can encode p110α, the catalytic subunit of phosphatidylinositol 3-kinases (PI3Ks). It is not only related to regulating various cellular physiological processes, such as cell survival motility, adhesion and apoptosis, but also exerts an guiding effect on cell growth, shape change and motility. Studies have shown that mutations of the PIK3CA gene mainly occur when the tumor is about to invade other tissues, which may lead to the enhanced lipid kinase activity, resulting in the out-of-control growth of cancer cells (6). In this case, a missense mutation within exon 7 in PIK3CA gene p.C420R could be found, which led to the change from T to C at base 1258, thereby inducing an alteration from cysteine to arginine at amino acid 420, while this contributes to the sustained PI3K/protein kinase B (AKT)/mammalian target of rapamycin (mTOR) pathway activation, thus promoting tumor development (7). Tumor cells with PIK3CA mutations are resistant to drugs targeting epidermal growth factor receptor (EGFR) or erythroblastic leukemia viral oncogene homolog 2 (ERBB2), but may be more sensitive to mTOR and PI3K inhibitors (8). Patients with PIK3CA gene p.C420R mutation can benefit from Alpelisib treatment (9). In light of this finding, PIK3CA is expected to be a therapeutic target for pleomorphic adenocarcinoma of the breast.

Four cases of pleomorphic adenocarcinoma of the breast were reported in relevant literature, among them, three were treated with radiation therapy even though no metastasis was seen in the lymph nodes following total mastectomy and sentinel lymph node exploration (10, 11). For the other one case, because the maximal tumor diameter was 10 cm, this patient was treated with neoadjuvant chemotherapy for 4 cycles, and the ultrasound examination showed that the tumor was significantly smaller than the previous one before performing total mastectomy and anterior sentinel lymph node exploration (4). In this study, the patient’s tumor was 3.3 cm in diameter, although there was no metastasis in the lymph nodes, the oncologist still recommended 4 courses of chemotherapy. Because the patient strongly refused systemic therapy, she chose to take only 1 course of oral Capecitabine after surgery. Therefore, there is no standardized treatment for pleomorphic adenocarcinoma of the breast, and a personalized plan needs to be made according to the patient’s situation.

Pleomorphic adenocarcinoma of the salivary gland is generally less invasive and is referred to as a low-grade pleomorphic adenocarcinoma in the salivary gland. However, some cases show aggressive biological behaviors, such as high-grade transformation, local recurrence, and distant metastasis (1). Due to the rarity of pleomorphic adenocarcinoma of the breast, its prognosis remains controversial. One 37-year-old case was reported in the literature, who developed liver metastases three years later and died in the same year due to extensive metastases despite a left breast nodule measuring only 1.5 cm and postoperative radiotherapy (10). In addition,TP53 gene mutation has been detected in pleomorphic adenocarcinoma of the breast, and PIK3CA gene mutation was identified in our patient. Both mutations are closely associated with poor prognosis in breast cancer patients, suggesting that pleomorphic adenocarcinoma of the breast may not be a low-grade malignant tumor (11). However, even though our patient only received one course of oral capecitabine after the operation, we followed up with the patient for two years after the operation, and the patient remained disease-free. It is similar to the other three cases reported (4, 10, 11). Based on our research and literature review, we are more inclined to consider that pleomorphic adenocarcinoma of the breast is a tumor with favorable prognosis. We will follow up and report on the patient’s further treatment.

## Conclusion

Breast pleomorphic adenocarcinoma has a very low prevalence, which can be definitely diagnosed based on characteristic morphologic manifestations with Bcl-2+. Breast pleomorphic adenocarcinoma may be a low-grade malignant tumor and may be spared from aggressive post-operative treatment even though the PIK3CA gene mutation can be identified in patients. Because the mutant allele frequency of PIK3CA was only 41.24%, which may impact its functionality and might not necessarily affect the clinical outcome. Moreover, more cases should be accumulated to have a deeper understanding of breast pleomorphic adenocarcinoma.

## Data Availability

The datasets presented in this study can be found in online repositories. The names of the repository/repositories and accession number(s) can be found in the article/**Supplementary Material**.
