# Supplementary material for: Pleomorphic adenocarcinoma of the breast: a case report
Source: Front Oncol. 2025 Sep 17;15:1644881. doi: 10.3389/fonc.2025.1644881 (PMC12483995; doi:10.3389/fonc.2025.1644881)
Supplement: Supplementary file 1 [file DataSheet1.zip › Table 1.DOCX]

**Supplementary Table 1.** The targeted NGS was conducted in 425 cancer-associated genes

| **No.** | **GENE** | **Genes related to targeted drugs** | **Chemotherapy-related genes** | **Genes related to genetic risks** |
| --- | --- | --- | --- | --- |
| 1 | ABCB1 (MDR1) | AKT1 | CDA | APC |
| 2 | ABCC2 (MRP2) | ALK | CYP2B6 | ATM |
| 3 | ADGRB3 (BAI3) | ARAF | DPYD | BMPR1A |
| 4 | ADH1B | ATM | ERCC1 | BRAF |
| 5 | AFDN (MLLT4) | BARD1 | ERCC2 | BRCA1 |
| 6 | AIP | BRAF | GSTP1 | BRCA2 |
| 7 | AKT1 | BRCA1 | MTHFR | BRIP1 |
| 8 | AKT2 | BRCA2 | NQO1 | CDH1 |
| 9 | AKT3 | BRIP1 | TPMT | CHEK2 |
| 10 | ALDH2 | CDK12 | TYMS | EGFR |
| 11 | ALK | CDK4 | UGT1A1 | EPCAM |
| 12 | AMER1 (FAM123B) | CDKN2A | XRCC1 | ERBB2 |
| 13 | APC | CHEK1 | TP53 | KIT |
| 14 | AR | CHEK2 | IDH1 | KRAS |
| 15 | ARAF | EGFR | IDH2 | MEN1 |
| 16 | ARID1A | ERBB2 | GSTM1 | MET |
| 17 | ARID1B | ESR1 | GSTT1 | MLH1 |
| 18 | ARID2 | EWSR1 | CYP2C9 | MSH2 |
| 19 | ARID5B | FANCL | CYP2C19 | MSH6 |
| 20 | ASCL4 | FGFR1 |  | MUTYH |
| 21 | ASXL1 | FGFR2 |  | NBN |
| 22 | ATF1 | FGFR3 |  | NF1 |
| 23 | ATIC | HRAS |  | NRAS |
| 24 | ATM | IDH1 |  | PALB2 |
| 25 | ATR | IDH2 |  | PIK3CA |
| 26 | ATRX | KIT |  | PMS2 |
| 27 | AURKA | KRAS |  | POLD1 |
| 28 | AURKB | MAP2K1 |  | POLE |
| 29 | AXIN2 | MDM2 |  | PTEN |
| 30 | AXL | MET |  | RAD51C |
| 31 | B2M | MTOR |  | RAD51D |
| 32 | BAD | NF1 |  | RB1 |
| 33 | BAK1 | NRAS |  | RET |
| 34 | BAP1 | NRG1 |  | SDHA |
| 35 | BARD1 | NTRK1 |  | SDHB |
| 36 | BAX | NTRK2 |  | SDHC |
| 37 | BCL2 | NTRK3 |  | SDHD |
| 38 | BCL2L11 (BIM) | PALB2 |  | SMAD4 |
| 39 | BCR | PDGFRA |  | STK11 |
| 40 | BIRC3 | PIK3CA |  | TP53 |
| 41 | BLM | PTCH1 |  | TSC1 |
| 42 | BMPR1AA62 | PTEN |  | TSC2 |
| 43 | BRAF | RAD51B |  | VHL |
| 44 | BRCA1 | RAD51C |  | FH |
| 45 | BRCA2 | RAD51D |  | CHEK1 |
| 46 | BRD4 | RAD54L |  | ATR |
| 47 | BRIP1 | RET |  | MRE11 |
| 48 | BTG2 | ROS1 |  | BARD1 |
| 49 | BTK | SMARCB1 |  | CDK4 |
| 50 | BUB1B | SMO |  | FANCL |
| 51 | CASP8 | TP53 |  | RAD54L |
| 52 | CBL | TSC1 |  | SMARCB1 |
| 53 | CBLB | TSC2 |  | AXIN2 |
| 54 | CCN6 (WISP3) |  |  | CDKN2A |
| 55 | CCND1 |  |  | FANCA |
| 56 | CCNE1 |  |  | FANCI |
| 57 | CD274 (PD-L1) |  |  | FLCN |
| 58 | CD74 |  |  | NF2 |
| 59 | CDA |  |  | PDGFRA |
| 60 | CDC73 |  |  | PRKAR1A |
| 61 | CDH1 |  |  | PTCH1 |
| 62 | CDK10 |  |  | WT1 |
| 63 | CDK12 |  |  | CDK12 |
| 64 | CDK4 |  |  | RAD51B |
| 65 | CDK6 |  |  |  |
| 66 | CDK8 |  |  |  |
| 67 | CDKN1A |  |  |  |
| 68 | CDKN1B |  |  |  |
| 69 | CDKN1C |  |  |  |
| 70 | CDKN2A |  |  |  |
| 71 | CDKN2B |  |  |  |
| 72 | CDKN2C |  |  |  |
| 73 | CEBPA |  |  |  |
| 74 | CEP57 |  |  |  |
| 75 | CHD4 |  |  |  |
| 76 | CHD8 |  |  |  |
| 77 | CHEK1 |  |  |  |
| 78 | CHEK2 |  |  |  |
| 79 | CREBBP |  |  |  |
| 80 | CRKL |  |  |  |
| 81 | CSF1R |  |  |  |
| 82 | CTCF |  |  |  |
| 83 | CTLA4 |  |  |  |
| 84 | CTNNB1 |  |  |  |
| 85 | CUL3 |  |  |  |
| 86 | CUX1 |  |  |  |
| 87 | CXCR4 |  |  |  |
| 88 | CYLD |  |  |  |
| 89 | CYP19A1 |  |  |  |
| 90 | CYP2A13 |  |  |  |
| 91 | CYP2A6 |  |  |  |
| 92 | CYP2A7 |  |  |  |
| 93 | CYP2B6*6 |  |  |  |
| 94 | CYP2C19*2 |  |  |  |
| 95 | CYP2C9*3 |  |  |  |
| 96 | CYP2D6 |  |  |  |
| 97 | CYP3A4*4 |  |  |  |
| 98 | CYP3A5 |  |  |  |
| 99 | CYSLTR2 |  |  |  |
| 100 | DAXX |  |  |  |
| 101 | DDR2 |  |  |  |
| 102 | DENND1A |  |  |  |
| 103 | DHFR |  |  |  |
| 104 | DICER1 |  |  |  |
| 105 | DLL3 |  |  |  |
| 106 | DNMT3A |  |  |  |
| 107 | DOT1L |  |  |  |
| 108 | DPYD |  |  |  |
| 109 | DTL (CDT2) |  |  |  |
| 110 | DUSP2 |  |  |  |
| 111 | EGFR |  |  |  |
| 112 | EIF1AX |  |  |  |
| 113 | EMSY (c11orf30) |  |  |  |
| 114 | EP300 |  |  |  |
| 115 | EPAS1 |  |  |  |
| 116 | EPCAM |  |  |  |
| 117 | EPHA2 |  |  |  |
| 118 | EPHA3 |  |  |  |
| 119 | EPHA5 |  |  |  |
| 120 | ERBB2 (HER2) |  |  |  |
| 121 | ERBB3 |  |  |  |
| 122 | ERBB4 |  |  |  |
| 123 | ERBIN (ERBB2IP) |  |  |  |
| 124 | ERCC1 |  |  |  |
| 125 | ERCC2 |  |  |  |
| 126 | ERCC3 |  |  |  |
| 127 | ERCC4 |  |  |  |
| 128 | ERCC5 |  |  |  |
| 129 | ESR1 |  |  |  |
| 130 | ETV1 |  |  |  |
| 131 | ETV4 |  |  |  |
| 132 | ETV5 |  |  |  |
| 133 | ETV6 |  |  |  |
| 134 | EWSR1 |  |  |  |
| 135 | EXT1 |  |  |  |
| 136 | EXT2 |  |  |  |
| 137 | EZH2 |  |  |  |
| 138 | EZR |  |  |  |
| 139 | FANCA |  |  |  |
| 140 | FANCC |  |  |  |
| 141 | FANCD2 |  |  |  |
| 142 | FANCE |  |  |  |
| 143 | FANCF |  |  |  |
| 144 | FANCG |  |  |  |
| 145 | FANCI |  |  |  |
| 146 | FANCL |  |  |  |
| 147 | FANCM |  |  |  |
| 148 | FAT1 |  |  |  |
| 149 | FBXW7 |  |  |  |
| 150 | FGF19 |  |  |  |
| 151 | FGFR1 |  |  |  |
| 152 | FGFR2 |  |  |  |
| 153 | FGFR3 |  |  |  |
| 154 | FGFR4 |  |  |  |
| 155 | FH |  |  |  |
| 156 | FLCN |  |  |  |
| 157 | FLT1 (VEGFR1) |  |  |  |
| 158 | FLT3 |  |  |  |
| 159 | FLT4 |  |  |  |
| 160 | FOXA1 |  |  |  |
| 161 | FOXL2 |  |  |  |
| 162 | FOXP1 |  |  |  |
| 163 | FRG1 |  |  |  |
| 164 | GATA1 |  |  |  |
| 165 | GATA2 |  |  |  |
| 166 | GATA3 |  |  |  |
| 167 | GATA4 |  |  |  |
| 168 | GATA6 |  |  |  |
| 169 | GNA11 |  |  |  |
| 170 | GNAQ |  |  |  |
| 171 | GNAS |  |  |  |
| 172 | GRIN2A |  |  |  |
| 173 | GRM3 |  |  |  |
| 174 | GRM8 |  |  |  |
| 175 | GSTM1 |  |  |  |
| 176 | GSTM4 |  |  |  |
| 177 | GSTP1 |  |  |  |
| 178 | GSTT1 |  |  |  |
| 179 | HDAC2 |  |  |  |
| 180 | HDAC9 |  |  |  |
| 181 | HGF |  |  |  |
| 182 | HLA-A |  |  |  |
| 183 | HNF1A |  |  |  |
| 184 | HNF1B |  |  |  |
| 185 | HRAS |  |  |  |
| 186 | IDH1 |  |  |  |
| 187 | IDH2 |  |  |  |
| 188 | IFNA6 |  |  |  |
| 189 | IFNB1 |  |  |  |
| 190 | IFNE |  |  |  |
| 191 | IFNG |  |  |  |
| 192 | IFNGR1 |  |  |  |
| 193 | IFNGR2 |  |  |  |
| 194 | IGF1R |  |  |  |
| 195 | IGF2 |  |  |  |
| 196 | IKBKE |  |  |  |
| 197 | IKZF1 |  |  |  |
| 198 | IL7R |  |  |  |
| 199 | INPP4B |  |  |  |
| 200 | IRF2 |  |  |  |
| 201 | JAK1 |  |  |  |
| 202 | JAK2 |  |  |  |
| 203 | JAK3 |  |  |  |
| 204 | JARID2 |  |  |  |
| 205 | JUN |  |  |  |
| 206 | KDM5A |  |  |  |
| 207 | KDR (VEGFR2) |  |  |  |
| 208 | KEAP1 |  |  |  |
| 209 | KIF1B |  |  |  |
| 210 | KIT |  |  |  |
| 211 | KITLG |  |  |  |
| 212 | KLLN |  |  |  |
| 213 | KMT2A (MLL) |  |  |  |
| 214 | KMT2B |  |  |  |
| 215 | KMT2C |  |  |  |
| 216 | KMT2D (MLL2) |  |  |  |
| 217 | KRAS |  |  |  |
| 218 | LHCGR |  |  |  |
| 219 | LMO1 |  |  |  |
| 220 | LRP1B |  |  |  |
| 221 | LYN |  |  |  |
| 222 | LZTR1 |  |  |  |
| 223 | MAP2K1 (MEK1) |  |  |  |
| 224 | MAP2K2 (MEK2) |  |  |  |
| 225 | MAP2K4 |  |  |  |
| 226 | MAP3K1 |  |  |  |
| 227 | MAP3K4 |  |  |  |
| 228 | MAX |  |  |  |
| 229 | MCL1 |  |  |  |
| 230 | MDM2 |  |  |  |
| 231 | MDM4 |  |  |  |
| 232 | MECOM |  |  |  |
| 233 | MED12 |  |  |  |
| 234 | MEF2B |  |  |  |
| 235 | MEN1 |  |  |  |
| 236 | MET |  |  |  |
| 237 | MGMT |  |  |  |
| 238 | MITF |  |  |  |
| 239 | MLH1 |  |  |  |
| 240 | MLH3 |  |  |  |
| 241 | MLLT1 |  |  |  |
| 242 | MLLT3 |  |  |  |
| 243 | MPL |  |  |  |
| 244 | MRE11 (MRE11A) |  |  |  |
| 245 | MSH2 |  |  |  |
| 246 | MSH6 |  |  |  |
| 247 | MTHFR |  |  |  |
| 248 | MTOR |  |  |  |
| 249 | MUTYH |  |  |  |
| 250 | MYC |  |  |  |
| 251 | MYCL (MYCL1) |  |  |  |
| 252 | MYCN |  |  |  |
| 253 | MYD88 |  |  |  |
| 254 | MYH9 |  |  |  |
| 255 | NAT1 |  |  |  |
| 256 | NBN |  |  |  |
| 257 | NCOR1 |  |  |  |
| 258 | NF1 |  |  |  |
| 259 | NF2 |  |  |  |
| 260 | NFE2L2 |  |  |  |
| 261 | NFKBIA |  |  |  |
| 262 | NKX2-1 |  |  |  |
| 263 | NOTCH1 |  |  |  |
| 264 | NOTCH2 |  |  |  |
| 265 | NOTCH3 |  |  |  |
| 266 | NPM1 |  |  |  |
| 267 | NQO1 |  |  |  |
| 268 | NRAS |  |  |  |
| 269 | NRG1 |  |  |  |
| 270 | NSD1 |  |  |  |
| 271 | NTRK1 |  |  |  |
| 272 | NTRK2 |  |  |  |
| 273 | NTRK3 |  |  |  |
| 274 | NUTM1 |  |  |  |
| 275 | PAK3 |  |  |  |
| 276 | PALB2 |  |  |  |
| 277 | PALLD |  |  |  |
| 278 | PARP1 |  |  |  |
| 279 | PARP2 |  |  |  |
| 280 | PAX5 |  |  |  |
| 281 | PBRM1 |  |  |  |
| 282 | PDCD1 (PD1) |  |  |  |
| 283 | PDCD1LG2 (PD-L2) |  |  |  |
| 284 | PDE11A |  |  |  |
| 285 | PDGFRA |  |  |  |
| 286 | PDGFRB |  |  |  |
| 287 | PDK1 |  |  |  |
| 288 | PGR |  |  |  |
| 289 | PHOX2B |  |  |  |
| 290 | PIK3C3 |  |  |  |
| 291 | PIK3CA |  |  |  |
| 292 | PIK3CD |  |  |  |
| 293 | PIK3R1 |  |  |  |
| 294 | PIK3R2 |  |  |  |
| 295 | PKHD1 |  |  |  |
| 296 | PLAG1 |  |  |  |
| 297 | PLCB4 |  |  |  |
| 298 | PLK1 |  |  |  |
| 299 | PMS1 |  |  |  |
| 300 | PMS2 |  |  |  |
| 301 | POLD1 |  |  |  |
| 302 | POLD3 |  |  |  |
| 303 | POLE |  |  |  |
| 304 | POLH |  |  |  |
| 305 | POT1 |  |  |  |
| 306 | PPARD |  |  |  |
| 307 | PPP2R1A |  |  |  |
| 308 | PRDM1 |  |  |  |
| 309 | PREX2 |  |  |  |
| 310 | PRF1 |  |  |  |
| 311 | PRKACA |  |  |  |
| 312 | PRKAR1A |  |  |  |
| 313 | PRKCI |  |  |  |
| 314 | PRKDC |  |  |  |
| 315 | PRKN (PARK2) |  |  |  |
| 316 | PRSS1 |  |  |  |
| 317 | PRSS3 |  |  |  |
| 318 | PTCH1 |  |  |  |
| 319 | PTEN |  |  |  |
| 320 | PTK2 |  |  |  |
| 321 | PTPN11 |  |  |  |
| 322 | PTPN13 |  |  |  |
| 323 | QKI |  |  |  |
| 324 | RAC1 |  |  |  |
| 325 | RAC3 |  |  |  |
| 326 | RAD50 |  |  |  |
| 327 | RAD51 |  |  |  |
| 328 | RAD51B |  |  |  |
| 329 | RAD51C |  |  |  |
| 330 | RAD51D |  |  |  |
| 331 | RAD54L |  |  |  |
| 332 | RAF1 |  |  |  |
| 333 | RARA |  |  |  |
| 334 | RARG |  |  |  |
| 335 | RASGEF1A |  |  |  |
| 336 | RB1 |  |  |  |
| 337 | RECQL4 |  |  |  |
| 338 | RELN |  |  |  |
| 339 | RET |  |  |  |
| 340 | RHOA |  |  |  |
| 341 | RICTOR |  |  |  |
| 342 | RNF43 |  |  |  |
| 343 | ROS1 |  |  |  |
| 344 | RPTOR |  |  |  |
| 345 | RRM1 |  |  |  |
| 346 | RUNX1 |  |  |  |
| 347 | RUNX1T1 |  |  |  |
| 348 | SBDS |  |  |  |
| 349 | SDC4 |  |  |  |
| 350 | SDHA |  |  |  |
| 351 | SDHB |  |  |  |
| 352 | SDHC |  |  |  |
| 353 | SDHD |  |  |  |
| 354 | SEPTIN9 (SEPT9) |  |  |  |
| 355 | SETBP1 |  |  |  |
| 356 | SETD2 |  |  |  |
| 357 | SF3B1 |  |  |  |
| 358 | SGK1 |  |  |  |
| 359 | SKP2 |  |  |  |
| 360 | SLC34A2 |  |  |  |
| 361 | SLC3A2 |  |  |  |
| 362 | SMAD2 |  |  |  |
| 363 | SMAD3 |  |  |  |
| 364 | SMAD4 |  |  |  |
| 365 | SMAD7 |  |  |  |
| 366 | SMARCA4 |  |  |  |
| 367 | SMARCB1 |  |  |  |
| 368 | SMO |  |  |  |
| 369 | SOCS1 |  |  |  |
| 370 | SOS1 |  |  |  |
| 371 | SOX2 |  |  |  |
| 372 | SPOP |  |  |  |
| 373 | SPRED1 |  |  |  |
| 374 | SPRY4 |  |  |  |
| 375 | SRC |  |  |  |
| 376 | SRSF2 |  |  |  |
| 377 | SRY |  |  |  |
| 378 | STAG2 |  |  |  |
| 379 | STAT3 |  |  |  |
| 380 | STK11 |  |  |  |
| 381 | STMN1 |  |  |  |
| 382 | SUFU |  |  |  |
| 383 | TACC3 |  |  |  |
| 384 | TAP1 |  |  |  |
| 385 | TAP2 |  |  |  |
| 386 | TEK |  |  |  |
| 387 | TEKT4 |  |  |  |
| 388 | TERC |  |  |  |
| 389 | TERT |  |  |  |
| 390 | TET2 |  |  |  |
| 391 | TGFBR2 |  |  |  |
| 392 | THADA |  |  |  |
| 393 | TMEM127 |  |  |  |
| 394 | TMPRSS2 |  |  |  |
| 395 | TNFAIP3 |  |  |  |
| 396 | TNFRSF11A |  |  |  |
| 397 | TNFRSF14 |  |  |  |
| 398 | TNFRSF19 |  |  |  |
| 399 | TNFSF11 |  |  |  |
| 400 | TOP1 |  |  |  |
| 401 | TOP2A |  |  |  |
| 402 | TP53 |  |  |  |
| 403 | TP63 |  |  |  |
| 404 | TPMT |  |  |  |
| 405 | TSC1 |  |  |  |
| 406 | TSC2 |  |  |  |
| 407 | TSHR |  |  |  |
| 408 | TTF1 |  |  |  |
| 409 | TUBB3 |  |  |  |
| 410 | TYMS |  |  |  |
| 411 | U2AF1 |  |  |  |
| 412 | UGT1A1 |  |  |  |
| 413 | VAMP2 |  |  |  |
| 414 | VEGFA |  |  |  |
| 415 | VHL |  |  |  |
| 416 | WAS |  |  |  |
| 417 | WRN |  |  |  |
| 418 | WT1 |  |  |  |
| 419 | XPA |  |  |  |
| 420 | XPC |  |  |  |
| 421 | XRCC1 |  |  |  |
| 422 | XRCC2 |  |  |  |
| 423 | YAP1 |  |  |  |
| 424 | ZNF217 |  |  |  |
| 425 | ZNF703 |  |  |  |
