# Supplementary material for: Pleomorphic adenocarcinoma of the breast: a case report
Source: Front Oncol. 2025 Sep 17;15:1644881. doi: 10.3389/fonc.2025.1644881 (PMC12483995; doi:10.3389/fonc.2025.1644881)
Supplement: Supplementary file 1 [file DataSheet1.zip › Table 2.docx]

**Table 2** The table of patient-related information.

| **Item** | **Details** |
| --- | --- |
| patient information | Female, 74 years old, Han ethnicity |
| main concerns and symptoms of the patient | Asymptomatic |
| medical, family, and psychosocial history | Hypertension and diabetes for 5 years; no psychiatric family history in three generations |
| relevant past interventions | Hypertension managed with amlodipine besylate PO; diabetes treated with metformin + gliclazide PO. (PO = per os/orally) |
